# Supplementary material for: A Novel Universal Primer Multiplex Real-Time PCR (UP-M-rtPCR) Approach for Specific Identification and Quantitation of Cat, Dog, Fox, and Mink Fractions Using Nuclear DNA Sequences
Source: Foods. 2023 Jan 31;12(3):594. doi: 10.3390/foods12030594 (PMC9914226; doi:10.3390/foods12030594)
Supplement: Supplementary file 1 [file foods-12-00594-s001.zip › Table S1.pdf]

Table S1. Cq values of 10-fold diluted cat, dog, fox, or mink DNA (5, 0.5, and 0.05 ng/μL).

Species	Concentration (ng/μL)	Average Cq ± SD	CV (%)
Cat	5	27.65 ± 0.32	1.16
	0.5	30.47 ± 0.10	0.33
	0.05	34.07 ± 0.03	0.09
Dog	5	28.47 ± 0.04	0.14
	0.5	31.6 ± 0.03	0.09
	0.05	34.84 ± 0.13	0.37
Fox	5	28.08 ± 0.10	0.35
	0.5	31.89 ± 0.15	0.47
	0.05	34.55 ± 0.16	0.46
Mink	5	27.17 ± 0.15	0.55
	0.5	31.23 ± 0.10	0.32
	0.05	34.03 ± 0.24	0.71
