# Supplementary material for: A Novel Universal Primer Multiplex Real-Time PCR (UP-M-rtPCR) Approach for Specific Identification and Quantitation of Cat, Dog, Fox, and Mink Fractions Using Nuclear DNA Sequences
Source: Foods. 2023 Jan 31;12(3):594. doi: 10.3390/foods12030594 (PMC9914226; doi:10.3390/foods12030594)
Supplement: Supplementary file 1 [file foods-12-00594-s001.zip › Table S2.pdf]

Table S2. Cq values of binary meat mixtures with different proportions of cat, dog, fox, or mink meat (5%, 0.5%, and 0.05%).

Species	Proportion (%)	Average Cq ± SD	CV (%)
Cat	5	26.96 ± 0.06	0.22
	0.5	29.95 ± 0.03	0.10
	0.05	33.67 ± 0.25	0.74
Dog	5	29.39 ± 0.06	0.20
	0.5	32.04 ± 0.10	0.31
	0.05	35.72 ± 0.09	0.25
Fox	5	29.51 ± 0.11	0.37
	0.5	32.36 ± 0.04	0.12
	0.05	35.73 ± 0.16	0.45
Mink	5	29.51 ± 0.03	0.10
	0.5	32.61 ± 0.03	0.09
	0.05	35.79 ± 0.09	0.25
